# Protein content and amino acid composition of commercially available plant-based protein isolates

**DOI:** 10.1007/s00726-018-2640-5

**Published:** 2018-08-30

**Authors:** Stefan H. M. Gorissen, Julie J. R. Crombag, Joan M. G. Senden, W. A. Huub Waterval, Jörgen Bierau, Lex B. Verdijk, Luc J. C. van Loon

**Affiliations:** 1 NUTRIM School of Nutrition and Translational Research in Metabolism, Maastricht University Medical Centre+, PO Box 616, 6200 MD Maastricht, The Netherlands; 2 Department of Clinical Genetics, Maastricht University Medical Centre+, Maastricht, The Netherlands

**Keywords:** Essential amino acid, Leucine, Plant-based protein, Muscle protein synthesis, Protein blend

## Abstract

The postprandial rise in essential amino acid (EAA) concentrations modulates the increase in muscle protein synthesis rates after protein ingestion. The EAA content and AA composition of the dietary protein source contribute to the differential muscle protein synthetic response to the ingestion of different proteins. Lower EAA contents and specific lack of sufficient leucine, lysine, and/or methionine may be responsible for the lower anabolic capacity of plant-based compared with animal-based proteins. We compared EAA contents and AA composition of a large selection of plant-based protein sources with animal-based proteins and human skeletal muscle protein. AA composition of oat, lupin, wheat, hemp, microalgae, soy, brown rice, pea, corn, potato, milk, whey, caseinate, casein, egg, and human skeletal muscle protein were assessed using UPLC–MS/MS. EAA contents of plant-based protein isolates such as oat (21%), lupin (21%), and wheat (22%) were lower than animal-based proteins (whey 43%, milk 39%, casein 34%, and egg 32%) and muscle protein (38%). AA profiles largely differed among plant-based proteins with leucine contents ranging from 5.1% for hemp to 13.5% for corn protein, compared to 9.0% for milk, 7.0% for egg, and 7.6% for muscle protein. Methionine and lysine were typically lower in plant-based proteins (1.0 ± 0.3 and 3.6 ± 0.6%) compared with animal-based proteins (2.5 ± 0.1 and 7.0 ± 0.6%) and muscle protein (2.0 and 7.8%, respectively). In conclusion, there are large differences in EAA contents and AA composition between various plant-based protein isolates. Combinations of various plant-based protein isolates or blends of animal and plant-based proteins can provide protein characteristics that closely reflect the typical characteristics of animal-based proteins.

## Introduction

Dietary protein intake stimulates muscle protein synthesis (Rennie et al. 1982). The muscle protein synthetic response to protein intake can vary substantially between different dietary protein types or sources. The differential muscle protein synthetic response is largely dependent on the postprandial availability of essential amino acids (and leucine in particular) to the muscle (Atherton et al. 2010; Volpi et al. 2003). Postprandial essential amino acid availability is regulated by a number of physiological processes including dietary protein digestion, amino acid absorption, splanchnic amino acid retention, and skeletal muscle perfusion (Groen et al. 2015) as well as various dietary factors including amino acid composition, essential amino acid content, and the presence of anti-nutritional factors.

Numerous studies have assessed the postprandial muscle protein synthetic response to the ingestion of dairy (Burd et al. 2012; Gorissen et al. 2016; Pennings et al. 2011, 2012; Tang et al. 2009; Witard et al. 2014; Yang et al. 2012a) and meat (Beals et al. 2016; Burd et al. 2015; Pennings et al. 2013; Symons et al. 2007, 2009, 2011; Phillips 2012; Robinson et al. 2013). The robust postprandial increase in muscle protein synthesis rates after the ingestion of these animal-based proteins is associated with the rapid rise in plasma essential amino acid concentrations, and leucine in particular. In comparison, the muscle protein synthetic responses to the ingestion of plant-based proteins such as soy (Phillips 2012; Tang et al. 2009; Wilkinson et al. 2007; Yang et al. 2012b) and wheat (Gorissen et al. 2016) have been shown to be of a lesser magnitude compared with animal-based proteins. The lesser anabolic properties of plant-based proteins have been attributed to lower essential amino acid content or shortage of specific amino acids such as leucine, lysine, and/or methionine (WHO/FAO/UNU Expert Consultation 2007; van Vliet et al. 2015; Young and Pellett 1994). All amino acids are required for protein synthesis, and lack of one or more amino acids may compromise the postprandial muscle protein synthetic response. Interestingly, the anabolic properties of plant-based proteins have only been studied for a few protein sources, such as soy (Fouillet et al. 2002, 2009; Hartman et al. 2007; Phillips 2012; Tang et al. 2009; Wilkinson et al. 2007; Yang et al. 2012b; Brown et al. 2004; Volek et al. 2013), wheat (Gorissen et al. 2016; Norton et al. 2009, 2012), and rice (Joy et al. 2013), despite the great diversity in plant-based protein sources (van Vliet et al. 2015).

The use of plant-based protein isolates in food formulations has recently become of interest due to greater sustainability and lower production costs. The current market offers a wide selection of plant-based proteins, but the lack of studies comparing plant-based proteins makes it difficult to select the most optimal plant-based proteins. We previously reported substantial differences in dietary protein characteristics between various plant-based protein sources (van Vliet et al. 2015). However, this report included data from a large number of research studies that used independent analyses and assessed only a single protein source or compared a few plant-based protein sources. In the current study, we applied the same analytical procedures on a large selection of commercially available protein isolates to provide a more comprehensive overview of the dietary protein characteristics of the main plant and animal-based protein isolates that are now widely available on the market.

In the present study, we characterized various plant-based protein isolates (oat, lupin, wheat, hemp, microalgae, soy, brown rice, pea, corn, and potato), animal-based protein isolates (whey, milk, caseinate, casein, and egg), and human skeletal muscle protein. Using ultra-performance liquid chromatography tandem mass spectrometry (UPLC–MS/MS), we assessed the amino acid composition of these protein types and sources. This study provides the basis for the identification of plant-based proteins with a high anabolic potential and for defining new plant-based protein blends that provide a complete spectrum of essential amino acids similar to most animal-based protein sources.

## Methods

### Protein sources

Thirty-five protein samples were selected that are presently commercially available as isolated protein powder suitable for application in human nutrition or animal feeds. Ten different plant-based protein sources including oat (*n *= 1), lupin (*n *= 1), wheat (*n *= 7), hemp (*n* = 1), microalgae (*n *= 1), soy (*n *= 7), brown rice (*n* = 1), pea (*n *= 3), corn (*n *= 3), and potato (*n *= 2) were compared with animal-based proteins including milk (*n *= 1), whey (*n *= 3), caseinate (*n *= 1), casein (*n *= 2), and egg (*n* = 1) as well as human skeletal muscle protein (*n *= 10). The plant-based protein sources selected for the current analyses account for approximately 67% of total plant-based protein intake, with oat providing 0.3%, wheat providing 32.3%, soy providing 2.7%, brown rice providing 20.6%, pea providing 1.0%, corn providing 7.3%, and potato providing 3.1% of total plant-based protein intake (FAOSTAT 2013). In addition, we included lupin, hemp, and microalgae in the current analyses. Lupin is a native European legume with a protein quality score similar to soy, and has become of interest as an alternative to the import of soy (Lucas et al. 2015; Mariotti et al. 2002). Microalgae have received considerable attention due to their high protein content (similar to meat, egg, soybean, and milk), presence of other beneficial nutrients, and a production that requires less water and land than other crops or animal foods (Bleakley and Hayes 2017). All protein samples were provided in kind by various suppliers: Agri Nutrition, Doetinchem, The Netherlands; Agridient, Hoofddorp, The Netherlands; Avebe, Veendam, The Netherlands; Cargill, Minnetonka, Minnesota, USA; Chamtor, Bazancourt, France; Cosucra, Warcoing, Belgium; FrieslandCampina DMV, Veghel, The Netherlands; FrieslandCampina Domo, Beilen, The Netherlands; L.I. Frank, Twello, The Netherlands; MRM Metabolic Response Modifiers, Oceanside, CA, USA; Roquette, Lestrem, France; Selecta, Goiânia-GO, Brazil; Tate and Lyle, Kimstad, Sweden; Tereos, Marckolsheim, France; Volac, Orwell, United Kingdom; Vitablend, Wolvega, The Netherlands; Wulro, Weert, The Netherlands. Protein samples were transported and stored in unopened packaging in a clean, dry, well-ventilated area at ambient temperature and humidity until further analysis. We included human skeletal muscle protein as reference protein with an ‘ideal’ amino acid composition when focusing on muscle protein synthesis. Human skeletal muscle samples were obtained from the *m. vastus lateralis* from ten volunteers who participated in a previously published trial (Gorissen et al. 2014). Protein samples were requested, obtained, and analyzed between December 2014 and June 2018.

### Protein content analysis

Approximately 10 mg of protein powder (in duplicate) or freeze-dried human skeletal muscle tissue was collected in steel crucibles. The Dumas combustion method was used to determine nitrogen using the vario MAX cube CN (Elementar Analysensysteme, Germany). Protein content was calculated by multiplying the determined nitrogen content by 6.25 as the standard nitrogen-to-protein conversion factor. There is an ongoing debate on the preferred use of protein source specific nitrogen-to-protein conversion factors that are known for some but not all of the protein sources included in the current analyses (Mariotti et al. 2008). In the present study, we used a single conversion factor (6.25) to enable direct comparisons between the various protein sources.

### Amino acid profile analysis

Approximately 6 mg of protein powder or freeze-dried human skeletal muscle tissue was hydrolyzed in 3 mL 6 M HCl for 12 h at 110 °C. After hydrolysis, samples were cooled down to 4 °C to stop the hydrolyzation process. HCl was evaporated under nitrogen stream and the dried amino acids were reconstituted in 5 mL water. Amino acid standards were obtained from Sigma-Aldrich (A9906) and diluted to final concentrations of 500, 375, 250, 125, 62.5, and 31.25 µM. 10 µL of the hydrolyzed protein sample or amino acid standard solution was mixed with 1500 µL 0.5 mM tridecafluoroheptanoic acid (TDFHA; Sigma) in water and 10 µL internal standard solution containing stable isotope-labeled amino acids (Cambridge Isotopes Laboratories) in 0.1 M HCl. Amino acid concentrations were determined using ultra-performance liquid chromatography (UPLC) tandem mass spectrometry (Waterval et al. 2009). Liquid chromatography was performed at 30 °C using a Acquity UPLC BEH C18, 1.7 μm, 2.1 × 100 mm column (Waters, Milford, MA, USA) and a gradient system with the mobile phase consisting of buffer A (0.5 mM TDFHA in water) and buffer B (0.5 mM TDFHA in acetonitrile) at a flow rate of 650 μL/min (split less). The gradient program used was: initial 99.5% A and 0.5% B; linear gradient to 70% A and 30% B in 14 min; hold for 3.5 min, return to initial conditions in 1 min at a flow rate of 700 μL/min, followed by equilibration for 10 min. One minute prior to the next sample injection the flow was set to 650 μL/min. Run-to-run time was 30 min. The injected volume was 5 μL. Mass spectrometry was performed using a Micromass Quattro Premier XE Tandem Mass Spectrometer (Waters, Milford, MA, USA). The mass spectrometer was used in the multiple reaction-monitoring mode (MRM) in the ESI-positive mode. The desolvation temperature was 450 °C, and the source temperature was 130 °C. The capillary voltage was set at 0.5 kV and the cone voltage was set at 25 V. Nitrogen gas was used as desolvation gas and as cone gas. Nitrogen gas was produced using an NM30L nitrogen generator (Peak Scientific, Renfrewshire, Scotland). The cone gas flow was 50 L/h and the desolvation gas flow was 800 L/h. Optimal detection conditions were determined by constant infusion of standard solutions (50 μM) in solvent A using a split system. MRM and daughter-ion scans were performed using argon as the collision gas at a pressure of 3.8 × 10^−3^ mbar and a flow of 0.2 mL/min.

During acid hydrolysis, the non-essential amino acids asparagine and glutamine are converted into aspartic acid and glutamic acid, respectively, and the essential amino acid tryptophan is decomposed, which precludes the ability to detect these amino acids (Fountoulakis and Lahm 1998). As tryptophan was not measured, the sum of essential amino acids includes threonine, methionine, phenylalanine, histidine, lysine, valine, isoleucine, and leucine. The acid hydrolysis was performed in the absence of oxygen and the hydrolyzation process was terminated after 12 h of incubation to minimize the reduction of cysteine and methionine. Although the acid hydrolysis is not optimal for all amino acids, we used this procedure for all protein samples to enable direct comparisons between the various protein sources.

## Results

### Protein content

**Fig. 1:**
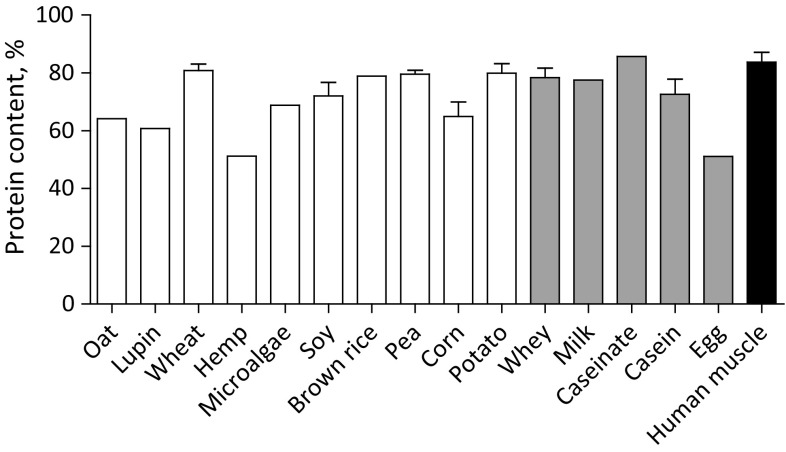
Mean (± SEM) protein content (% of raw material) of various dietary protein sources and human skeletal muscle tissue based on the determined nitrogen content multiplied by 6.25 as the standard conversion factor. White bars represent plant-based protein sources, grey bars represent animal-derived protein sources, and black bar represents human skeletal muscle protein

Protein contents ranged between 51 and 86% of raw material (Fig. 1). Plant-based protein sources ranged between 51 and 81% and protein content was lower in hemp (51%), lupin (61%), oat (64%), and corn (65%) and higher in brown rice (79%), pea (80%), potato (80%), and wheat (81%). The protein content of animal-based proteins ranged between 51% in egg and 86% in calcium caseinate. Freeze-dried human skeletal muscle tissue contained 84% protein. Protein contents of various samples from the same protein source differed between suppliers, with protein contents of wheat protein ranging from 74 to 88%, soy protein ranging from 61 to 91%, pea protein ranging from 77 to 81%, corn protein ranging from 58 to 75%, potato protein ranging from 77 to 83%, whey protein ranging from 72 to 84%, and casein ranging from 67 to 78%.

### Essential amino acid content

**Fig. 2:**
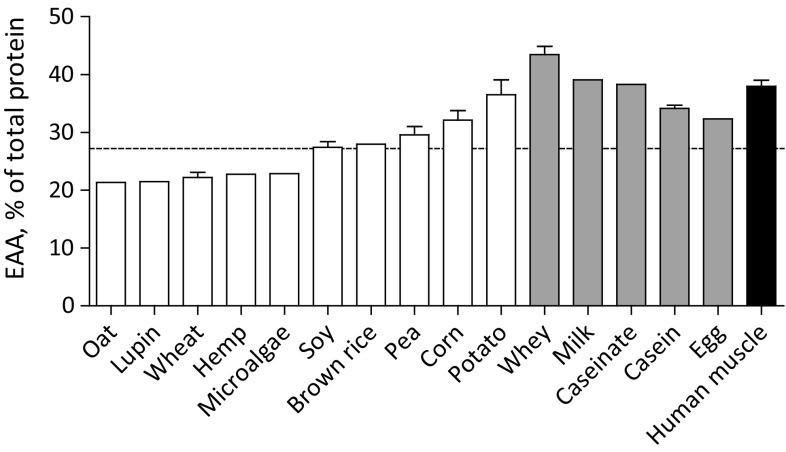
Mean (± SEM) essential amino acid (EAA) contents (% of total protein) of various dietary protein sources and human skeletal muscle protein. White bars represent plant-based protein sources, grey bars represent animal-derived protein sources, and black bar represents human skeletal muscle protein. Dashed line represents the amino acid requirements for adults (WHO/FAO/UNU Expert Consultation 2007). Note: EAA is the sum of His, Ile, Leu, Lys, Met, Phe, Thr, and Val. Trp was not measured

Essential amino acid contents are shown in Fig. 2. Essential amino acid contents were lower in plant-based (26 ± 2% of total protein) when compared with animal-based proteins (37 ± 2% of total protein) and human skeletal muscle protein (38% of total protein). The essential amino acid contents of the plant-based proteins oat (21%), lupin (21%), wheat (22%), hemp (23%), and microalgae (23%) are below the WHO/FAO/UNU amino acid requirements (WHO/FAO/UNU Expert Consultation 2007). Thus, the essential amino acid requirement would not be met when one of these proteins would be the only protein source consumed. Note that the requirement is based on a recommended adult protein intake of 0.66 g/kg body weight/day. Plant-based proteins that do meet the requirements for essential amino acids include soy (27%), brown rice (28%), pea (30%), corn (32%), and potato (37%). Of the animal-based proteins, whey protein had the highest essential amino acid content of 43%. Milk protein (39%) and calcium caseinate (38%) showed an intermediate and casein (34%) and egg (32%) a lower essential amino acid content. All animal-based proteins were well above the WHO/FAO/UNU amino acid requirements.

### Amino acid profiles

**Fig. 3:**
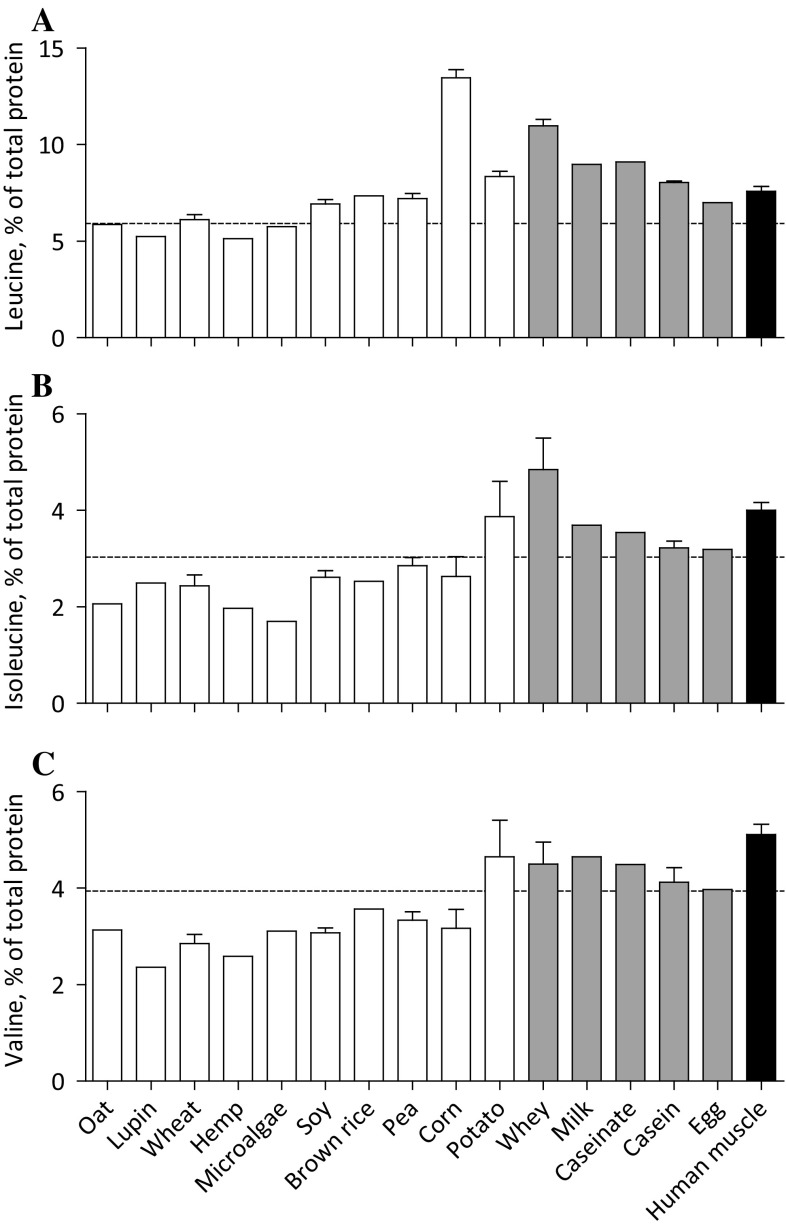
Mean (± SEM) leucine (**a**), isoleucine (**b**), and valine (**c**) contents (% of total protein) of various dietary protein sources and human skeletal muscle protein. White bars represent plant-based protein sources, grey bars represent animal-derived protein sources, and black bar represents human muscle. Dashed line represents the amino acid requirements for adults (WHO/FAO/UNU Expert Consultation 2007)

**Fig. 4:**
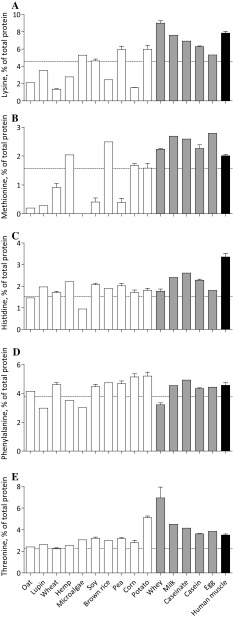
Mean (± SEM) lysine (**a**), methionine (**b**), histidine (**c**), phenylalanine (**d**), and threonine (**e**) contents (% of total protein) of various dietary protein sources and human skeletal muscle protein. White bars represent plant-based protein sources, grey bars represent animal-derived protein sources, and black bar represents human muscle. Dashed line represents the amino acid requirements for adults (WHO/FAO/UNU Expert Consultation 2007)

Amino acid profiles differed substantially among plant-based proteins with leucine contents as low as 5.1% in hemp, 5.2% in lupin, and 5.8% in microalgae and as high as 13.5% in corn and 8.3% in potato compared to 7.6% in human skeletal muscle protein (Fig. 3a). Despite the high leucine content of corn and potato, the average leucine content of plant-based proteins was lower (7.1 ± 0.8%) when compared with animal-based proteins (8.8 ± 0.7%). Lysine and methionine contents are particularly low in plant-based proteins (3.6 ± 0.6 and 1.0 ± 0.3%, respectively) when compared with animal-based proteins (7.0 ± 0.6 and 2.5 ± 0.1%, respectively) and human skeletal muscle protein (7.8 and 2.0%, respectively), but with great variability between plant-based protein sources (Fig. 4a, b). The lysine content of wheat (1.4%), corn (1.5%), oat (2.1%), brown rice (2.4%), hemp (2.8%), and lupin (3.5%) is below the WHO/FAO/UNU requirements and substantially lower when compared with soy (4.6%), microalgae (5.3%), pea (5.9%), and potato (6.0%). Methionine contents were low in microalgae (0.0%), oat (0.2%), lupin (0.3%), pea (0.4%), soy (0.4%), and wheat (0.9%), but reached the WHO/FAO/UNU requirements in potato (1.6%), corn (1.7%), hemp (2.0%), and brown rice (2.5%). Less pronounced variability was observed between plant-based and animal-based proteins in isoleucine, valine, histidine, phenylalanine, and threonine contents. Except for potato protein, the contents of the branched-chain amino acids isoleucine and valine were lower in plant-based when compared to animal-based proteins and did not reach the WHO/FAO/UNU requirements. A complete overview of the amino acid profiles as expressed in g/100 g raw material is presented in Table 1.

**Table 1:** Amino acid content of various dietary protein sources and human skeletal muscle

	Oat	Lupin	Wheat	Hemp	Microalgae	Soy	Brown rice	Pea	Corn	Potato	Whey	Milk	Caseinate	Casein	Egg	Human muscle
Essential amino acids																
Threonine	1.5	1.6	1.8	1.3	2.1	2.3	2.3	2.5	1.8	4.1	5.4	3.5	3.5	2.6	2.0	2.9
Methionine	0.1	0.2	0.7	1.0	0.0	0.3	2.0	0.3	1.1	1.3	1.8	2.1	2.2	1.6	1.4	1.7
Phenylalanine	2.7	1.8	3.7	1.8	2.1	3.2	3.7	3.7	3.4	4.2	2.5	3.5	4.2	3.1	2.3	3.8
Histidine	0.9	1.2	1.4	1.1	0.7	1.5	1.5	1.6	1.1	1.4	1.4	1.9	2.2	1.7	0.9	2.8
Lysine	1.3	2.1	1.1	1.4	3.6	3.4	1.9	4.7	1.0	4.8	7.1	5.9	5.9	4.6	2.7	6.6
Valine	2.0	1.4	2.3	1.3	2.1	2.2	2.8	2.7	2.1	3.7	3.5	3.6	3.8	3.0	2.0	4.3
Isoleucine	1.3	1.5	2.0	1.0	1.2	1.9	2.0	2.3	1.7	3.1	3.8	2.9	3.0	2.3	1.6	3.4
Leucine	3.8	3.2	5.0	2.6	4.0	5.0	5.8	5.7	8.8	6.7	8.6	7.0	7.8	5.8	3.6	6.3
ΣEAA	13.7	13.1	18.0	11.6	15.7	19.9	22.1	23.6	21.0	29.3	34.1	30.3	32.8	24.8	16.5	31.8
Non-essential amino acids																
Serine	2.2	2.5	3.5	2.3	2.1	3.4	3.4	3.6	2.9	3.4	4.0	4.0	4.2	3.4	3.3	2.3
Glycine	1.7	2.1	2.4	2.1	2.6	2.7	3.4	2.8	1.6	3.2	1.5	1.5	1.5	1.2	1.4	3.1
Glutamic acid	11.0	12.4	26.9	7.4	5.7	12.4	12.7	12.9	13.1	7.1	15.5	16.7	16.0	13.9	5.1	13.1
Proline	2.5	2.0	8.8	1.8	2.3	3.3	3.4	3.1	5.2	3.3	4.8	7.3	8.7	6.5	1.8	0.0
Cysteine	0.4	0.2	0.7	0.2	0.1	0.2	0.6	0.2	0.3	0.3	0.8	0.2	0.1	0.1	0.4	0.0
Alanine	2.2	1.7	1.8	1.9	4.0	2.8	4.3	3.2	4.8	3.3	4.2	2.6	2.6	2.0	2.6	4.1
Tyrosine	1.5	1.9	2.4	1.3	1.2	2.2	3.5	2.6	2.7	3.8	2.4	3.8	4.4	3.4	1.8	2.0
Arginine	3.1	5.5	2.4	5.3	3.4	4.8	5.4	5.9	1.7	3.3	1.7	2.6	2.9	2.1	2.6	4.4
ΣNEAA	24.7	28.2	48.9	22.4	21.4	31.9	36.8	34.4	32.3	27.8	34.9	38.6	40.4	32.5	19.0	29.0

Values are presented in g per 100 g raw material. Tryptophan, aspartic acid, asparagine, and glutamine were not measured

*ΣEAA* sum of all essential amino acids, *ΣNEAA* sum of all non-essential amino acids

### Relative protein intake

Table 2 shows representative amounts of protein or raw material that need to be consumed to allow 2.7 g of leucine or 10.9 g essential amino acids to be ingested, which is the amount of leucine or essential amino acids present in 25 g whey protein that has been shown to stimulate muscle protein synthesis in humans (Gorissen et al. 2017; Mitchell et al. 2015; Witard et al. 2014; Yang et al. 2012a). Between 20 and 54 g of plant-based protein needs to be consumed to ingest 2.7 g leucine, which would be provided by, e.g., 31 g corn protein powder or 105 g hemp protein powder. This again highlights the variability among plant-based protein sources.

**Table 2:** Representative amount of protein

	Matched for leucine		Matched for ΣEAA	
	Amount of protein (g)	Amount of raw material (g)	Amount of protein (g)	Amount of raw material (g)
Oat	47	73	51	79
Lupin	52	86	50	83
Wheat	45	55	49	60
Hemp	54	105	48	93
Microalgae	48	69	48	69
Soy	40	55	40	55
Brown rice	37	47	39	49
Pea	38	48	37	46
Corn	20	31	34	52
Potato	33	41	30	37
Whey	25	32	25	32
Milk	31	39	28	36
Caseinate	30	35	28	33
Casein	34	47	32	44
Egg	39	77	34	66

Amount of a certain protein source that needs to be consumed to provide 2.7 g leucine or 10.9 g essential amino acids (i.e., the same amount of leucine or essential amino acids ingested when consuming 25 g whey protein)

*ΣEAA* sum of all essential amino acids

## Discussion

This study measures and compares amino acid composition of various plant-based protein isolates including oat, lupin, wheat, hemp, microalgae, soy, brown rice, pea, corn, and potato with animal-derived proteins and human skeletal muscle protein. We observed that plant-based proteins have relatively low essential amino acid and leucine contents when compared with animal-based proteins and human skeletal muscle protein. In addition, some but not all plant-based protein isolates are low in lysine and/or methionine contents. As there is a large variability in amino acid composition among the various plant-based protein sources, a balanced combination of different plant-based proteins may provide a high(er) quality protein blend.

World population growth in combination with increasingly limited resources (arable land and fresh water) has resulted in the need for alternative protein sources to meet global protein requirements. The production of plant-based foods requires less land and water and is associated with lower greenhouse gas emissions compared with animal-based foods. However, studies suggest that plant-based proteins are of lesser quality with respect to their ability to increase postprandial muscle protein synthesis rates. This belief is mainly based on the very few studies that assessed the muscle protein synthetic response to the ingestion of soy protein (Phillips 2012; Tang et al. 2009; Wilkinson et al. 2007; Yang et al. 2012b). Lower essential amino acid contents and/or relative shortage of leucine, lysine, and/or methionine in the protein may contribute to the lower anabolic capacity of plant-based proteins. However, there is a large variability in amino acid composition among different plant-based protein sources. In a recent literature review, we compared data on essential amino acid, leucine, lysine, and methionine contents between various plant and animal-based protein sources (van Vliet et al. 2015). This review used data obtained from a large selection of publications that applied many different analytical procedures to assess protein characteristics such as nitrogen content and amino acid composition. In the current study, we collected a large number of commercially available dietary protein powders and applied the same analytical procedures on all protein sources, including the Dumas combustion method to determine the protein content of the protein powder (Jung et al. 2003) and ultra-performance liquid chromatography tandem mass spectrometry to assess the amino acid composition of the protein sources (Waterval et al. 2009). Consequently, we compared protein contents as well as essential and non-essential amino acid contents and, more specifically, leucine, lysine, and methionine contents between different plant-based proteins, animal-based proteins, and human skeletal muscle protein.

Of the amino acids, the essential amino acids seem to be primarily responsible for the postprandial stimulation of muscle protein synthesis (Tipton et al. 1999a, b; Volpi et al. 2003). There is a dose-dependent relationship between the amount of essential amino acids ingested and the postprandial muscle protein synthetic response until a plateau is reached (Cuthbertson et al. 2005). When identifying dietary protein sources that could effectively be used in dietary interventions to promote muscle growth or prevent muscle loss, it is important to consider the essential amino acid content of the dietary protein source. Although we observed that the average essential amino acid contents of plant-based proteins are generally lower when compared with animal-based proteins and human skeletal muscle protein, certain plant-based proteins have a relatively high essential amino acid content. Soy, brown rice, pea, corn, and potato protein have essential amino acid contents that meet the requirements as recommended by the WHO/FAO/UNU (WHO/FAO/UNU Expert Consultation 2007) (Fig. 2). In addition, the essential amino acid content of potato protein (37%) is in fact greater when compared with casein (34%) and egg (32%). These data suggest that certain plant-based proteins could theoretically provide sufficient essential amino acids to allow a robust postprandial stimulation of muscle protein synthesis.

Upon digestion and absorption, dietary protein provides amino acids that serve as precursor for de novo muscle protein synthesis. Besides serving as precursors for de novo protein synthesis, certain amino acids can directly activate the muscle protein synthetic machinery by activating mTORC1 and downstream anabolic signaling (Atherton et al. 2010). Specifically, leucine has been shown to be sensed by Sestrin2 that promotes translocation of mTORC1 to the lysosomal membrane, where it becomes activated (Laplante and Sabatini 2012; Saxton et al. 2016; Wolfson et al. 2016), leading to activation of downstream signaling and subsequent stimulation of muscle protein synthesis. As such, the leucine content of the ingested protein source forms a key characteristic that modulates activation of the muscle protein synthetic machinery after protein ingestion. In this regard, we observed that hemp (5.1% leucine) and lupin (5.2% leucine) do not meet the WHO/FAO/UNU requirements for leucine of 5.9% (WHO/FAO/UNU Expert Consultation 2007), whereas microalgae, oat, and wheat provide close to the leucine requirements, and soy, pea, brown rice, potato, and corn provide well above the leucine requirements (Fig. 3a). Interestingly, the leucine content of potato (8.3%) is greater when compared with casein (8.0%) and egg (7.0%) and the leucine content of corn (13.5%) is greater than whey (11.0%) protein (compared to 7.6% in human skeletal muscle protein). It has been well established that the ingestion of 25 g whey protein (providing 2.7 g leucine) results in a robust stimulation of muscle protein synthesis rates (Gorissen et al. 2017; Mitchell et al. 2015; Witard et al. 2014; Yang et al. 2012a). Plant-based proteins could provide the same amount of leucine by adjusting the amount of protein ingested. Due to the greater leucine content of corn, ‘merely’ 20 g of protein needs to be ingested to provide 2.7 g leucine, while the dietary protein dose of the other plant-based proteins would need to be increased to 33 g (potato), 37 g (brown rice), 38 g (pea), 40 g (soy), 45 g (wheat), 47 g (oat), 48 g (microalgae), 52 g (lupin), and 54 g (hemp) (Table 2). Ingesting these amounts of protein may be sufficient to activate the muscle protein synthetic machinery, assuming that 2.7 g leucine is sufficient to trigger this activation. Once activated, all amino acids are required to serve as precursors for de novo tissue protein synthesis and shortage of one or more specific amino acids could compromise a sustained elevation in postprandial muscle protein synthesis rates.

The lysine and methionine contents are generally low(er) in plant-based when compared with animal-derived proteins (WHO/FAO/UNU Expert Consultation 2007; van Vliet et al. 2015; Young and Pellett 1994). The current analysis confirms these findings and shows that methionine and lysine contents are lower in plant-based proteins (1.0 ± 0.3 and 3.6 ± 0.6%) compared with animal-based proteins (2.5 ± 0.1 and 7.0 ± 0.6%) and human skeletal muscle protein (2.0 and 7.8%, respectively). Interestingly, we observed a greater variability among the plant-based proteins, with some plant-based proteins providing the requirements of lysine (4.5%) and others providing the requirements of methionine (1.6%). More specifically, soy, microalgae, and pea contain 4.6, 5.3, and 5.9% lysine, respectively, but are low in methionine. Alternatively, corn, hemp, and brown rice contain 1.7, 2.0, and 2.5% methionine, respectively, but are low in lysine (Fig. 4a, b). Oat, lupin, and wheat protein are low in both lysine and methionine, whereas potato protein contains sufficient levels of both lysine (6.0%) and methionine (1.6%).

Studies investigating the anabolic properties of plant-based proteins have shown that the muscle protein synthetic response to the ingestion of soy (Tang et al. 2009; Wilkinson et al. 2007; Yang et al. 2012b) and wheat protein (Gorissen et al. 2016) is lower when compared with dairy protein. In an attempt to increase the muscle protein synthetic response to soy protein, Yang and colleagues (Yang et al. 2012b) increased the protein dose from 20 to 40 g, but the ingestion of this higher dose of soy protein was not able to induce a greater stimulation of muscle protein synthesis. We recently showed that increasing the amount of wheat protein hydrolysate from 35 to 60 g, thereby matching for the leucine content of 35 g whey protein, was able to substantially increase postprandial muscle protein synthesis rates (Gorissen et al. 2016). Although effective, simply increasing the dose of plant-based proteins to compensate for their lower anabolic properties may not always be practical or feasible. Of the ten plant-based proteins included in the current analysis, potato protein is the only protein source containing the WHO/FAO/UNU requirements for all essential amino acids. Thus, when consuming potato protein as the only dietary protein source at the recommended adult protein intake level of 0.66 g/kg/day, sufficient amounts of all essential amino acids should be consumed. It remains to be investigated whether the ingestion of a single meal-like amount of potato protein has the capacity to stimulate muscle protein synthesis. The other nine plant-based protein isolates included in the current analysis contain insufficient amounts of lysine and/or methionine according to the WHO/FAO/UNU requirements. The low lysine or methionine content of corn, hemp, brown rice, soy, and pea protein can be compensated for by ingesting 2–4 times more protein. Alternatively, combining corn, hemp, or brown rice (low in lysine) with soy, microalgae, or pea (low in methionine) at a 50/50 ratio results in protein blends with a more ‘complete’ amino acid composition. These blends contain intermediate amounts of lysine and methionine and require only 10–90% more protein to be consumed to provide sufficient amounts of all essential amino acids (instead of the 2–4 times higher dose when a single protein source would be consumed). Oat, lupin, and wheat protein are low in both lysine and methionine, which could be compensated for by ingesting 3–8 times more protein. However, a more realistic approach would be to combine oat, lupin, or wheat protein with animal-based proteins. At a 50/50 ratio, these blends require only 5–40% more protein to be consumed to provide sufficient amounts of all essential amino acids. Certainly, many more protein blends combining two or more protein sources at various ratios could be created. Creating protein blends seems to offer benefits over increasing the dose of protein consumed, as protein blends can provide sufficient amounts of all essential amino acids at a lower protein dose. Whether the ingestion of a single meal-size bolus of these protein blends increases muscle protein synthesis rates remains to be assessed. Promising results have been obtained in both young (Reidy et al. 2013, 2014, 2016) and older individuals (Borack et al. 2016) when using a protein blend composed of 50% caseinate, 25% whey protein, and 25% soy protein. Future studies should assess the anabolic properties of protein blends with a greater relative amount of plant-based proteins, protein blends composed of solely plant-based proteins designed to provide a more balanced essential amino acid profile, and/or individual plant-based proteins with a more optimal amino acid composition. Dietary intervention can then implement plant-based proteins or protein blends with greater anabolic properties as a more sustainable protein source to meet global protein requirements and support overall growth, health, as well as muscle mass maintenance throughout the lifespan.

In conclusion, there are large differences in amino acid content and amino acid composition between the various plant-based protein sources. Combinations of various plant-based protein sources or blends of animal- and plant-based proteins may provide protein characteristics that closely reflect the typical characteristics of animal-based protein sources.
